# Asymmetric Multisensory Interactions of Visual and Somatosensory Responses in a Region of the Rat Parietal Cortex

**DOI:** 10.1371/journal.pone.0063631

**Published:** 2013-05-07

**Authors:** Michael T. Lippert, Kentaroh Takagaki, Christoph Kayser, Frank W. Ohl

**Affiliations:** 1 Department Systems Physiology of Learning, Leibniz Institute for Neurobiology, Magdeburg, Germany; 2 Departmen Physiology of Cognitive Processes, Max Planck Institute for Biological Cybernetics, Tübingen, Germany; 3 Institute of Neuroscience and Psychology, University of Glasgow, Glasgow, United Kingdom; 4 Institute of Biology, Otto-von-Guericke University Magdeburg, Germany; 5 Center for Behavioral Brain Sciences, Magdeburg, Germany; Instituto de Neurociencias de Alicante UMH-CSIC, Spain

## Abstract

Perception greatly benefits from integrating multiple sensory cues into a unified percept. To study the neural mechanisms of sensory integration, model systems are required that allow the simultaneous assessment of activity and the use of techniques to affect individual neural processes in behaving animals. While rodents qualify for these requirements, little is known about multisensory integration and areas involved for this purpose in the rodent. Using optical imaging combined with laminar electrophysiological recordings, the rat parietal cortex was identified as an area where visual and somatosensory inputs converge and interact. Our results reveal similar response patterns to visual and somatosensory stimuli at the level of current source density (CSD) responses and multi-unit responses within a strip in parietal cortex. Surprisingly, a selective asymmetry was observed in multisensory interactions: when the somatosensory response preceded the visual response, supra-linear summation of CSD was observed, but the reverse stimulus order resulted in sub-linear effects in the CSD. This asymmetry was not present in multi-unit activity however, which showed consistently sub-linear interactions. These interactions were restricted to a specific temporal window, and pharmacological tests revealed significant local intra-cortical contributions to this phenomenon. Our results highlight the rodent parietal cortex as a system to model the neural underpinnings of multisensory processing in behaving animals and at the cellular level.

## Introduction

The question of how the brain integrates multisensory information into a unified percept has received much recent attention [1]–[4]. A number of cortical areas have been implicated in this process, ranging from primary sensory areas to classical association cortices in the temporal, frontal, and parietal lobes [5]–[7]. In primary sensory areas, subtle modulatory influences via stimulation of other sensory modalities predominate [8]–[11], while 'higher' association regions show a higher prevalence of multisensory neurons compared to primary regions [12]–[15].

Studies in the primate brain provided pioneering insights into this question. For example, functional imaging studies have linked human perception with large-scale sensory parcellation of the brain [16]–[18], and microelectrode recordings have elucidated specific multisensory coding strategies in individual cortical areas [2], [4], [11], [19]–[22]. However, causal mechanistic understanding of this integration process requires investigation in a model system that allows i) simultaneous high-resolution assessment of neural activity across sensory streams, ii) the use of genetic techniques to manipulate or interfere with normal function, and iii) ideally, the possibility to perform these methods in awake behaving animals. Given the current state of technological advances in optogenetic techniques [23] and behavioral model systems [24], [25], rodents are ideally suited to address these requirements [23], [25]–[28].

Aside from the key technical advantages of rodent preparations, inquiry into both rat cognition in and of itself and into the study of its underlying neural bases have also gained considerable interest in recent years [29]–[32]. In the parietal cortex of rat, previous studies clearly suggest the existence of multisensory responses [33]–[35]. These results are further supported by work done in primate and ferret parietal cortex [20], [36]–[38] and in human parietal cortex [39]–[41]. Furthermore, the parietal cortex is an association area with a strong influence on behavior [40], [42], [43]. Rats are known to combine visual and somatosensory information to perform tasks [44]–[47], and show performance benefits similar to those in humans [47], [48]. Whether such multisensory performance benefits rely on the parietal cortex is an interesting question. Several investigators reported that multisensory neurons prevail at the border between visual, auditory, and somatosensory cortical areas in the rat [33], [49], [50] and implicated the parietal region in multisensory processing [20], [22], [39], [41], [51]. For example, Toldi and colleagues reported evoked potentials to visual and somatosensory stimuli in a graded multisensory zone between visual and somatosensory regions, and described sub-additive interactions between individual evoked potentials [49].

To investigate multisensory integration, early studies in the cat superior colliculus derived three basic rules of multisensory integration: a spatial rule predicting higher efficiency of interaction for spatially congruent stimuli, a temporal rule postulating the same for temporal alignment and a rule of inverse effectiveness [52]–[54]. The latter predicts stronger modulations of activities if at least one of the stimuli is weak. Whether such properties prevail in rat parietal cortex is unclear, and if they do, their underlying response properties and neural mechanisms remain to be investigated.

Here, we employed a combination of optical imaging, laminar electrophysiology, and pharmacological manipulations to localize and investigate multisensory interactions in the rat parietal cortex. One aspect which highlights the parietal cortex as an area for multisensory integration is its localization between the primary visual cortex and somatosensory cortex, and especially the barrel sub-field of the latter. This spatial relationship could hypothetically enable direct interaction of unisensory responses via intracortically propagating activity along horizontal connections as predicted by earlier works [34], [35]. Such local cortical interactions will, on the one hand, add to the understanding of multisensory integration and also contribute to the understanding of general cortical mass action. In order to maximize the chance for such local interactions, we stimulated with full-field light flashes and whole-field whisker deflections, rather than focusing on precise receptive field relations of individual neurons [33].

We concentrate on non-linear interactions between responses to different stimuli as one hallmark of multisensory integration, that is, deviations of the response to the multisensory stimulus from the sum of respective unisensory activities [1], [55]. Given linear superposition of electrical fields, a violation of this linearity principle indicates neuronal interactions. Furthermore, we investigate the timing of such interactions, since the temporal rule predicts interactions only for stimuli spaced closely in time.

## Materials and Methods

### Animals and Anesthesia

All experiments were in compliance with the guidelines of the European Community (EUVD 86/609/EEC) and were approved by the ethics commission of the state of Sachsen-Anhalt (Landesverwaltungsamt Halle). Fourteen adult male Wistar rats were used (11 for parietal and three for primary areas). A jugular venous catheter for anesthetic induction was implanted at least 2 days prior to recordings. On the day of the experiment, animals were pretreated with 0.2 mg/kg glycopyrrolate (Robinul, Riemser AG) to reduce mucous secretion. Anesthesia was induced intravenously using an aqueous solution of urethane (ethyl carbamate, Sigma-Aldrich, 0.625 g/ml) until a concentration of 1.5 g/kg body weight was reached [56]. Anesthetic depth was monitored, and supplements of urethane were administered to achieve areflexia. Local anesthetic (Lidocaine, AstraZeneca) was applied. This procedure resulted in a reproducible, moderate stage of anesthesia.

### Optical Imaging

**Figure 1:**
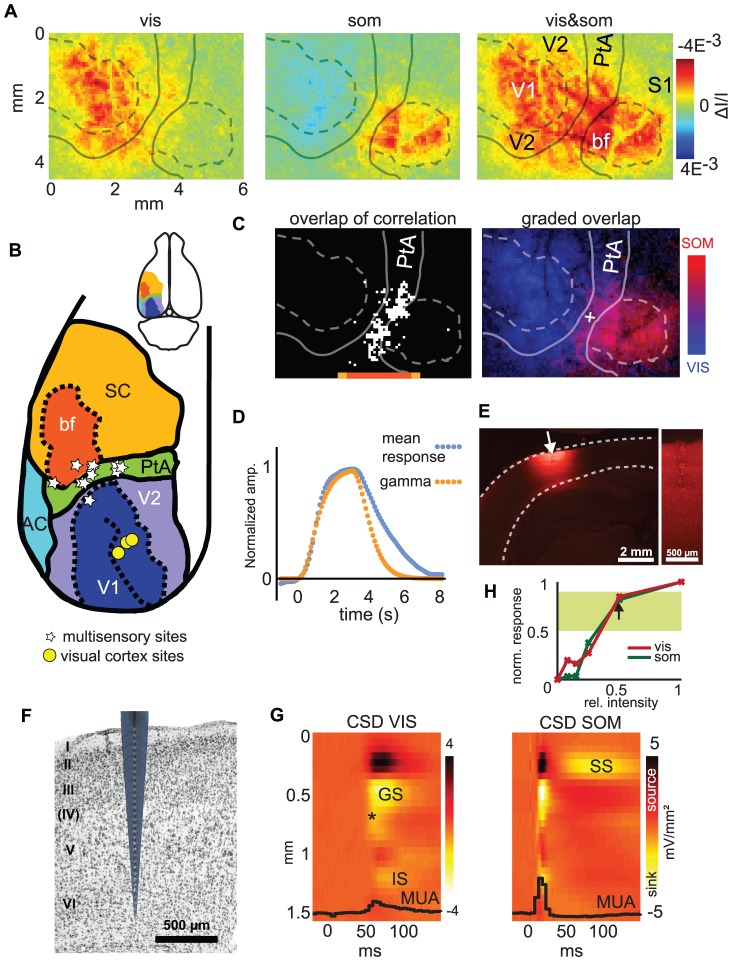
Functional localization of a multisensory parietal area. A: Intrinsic optical imaging was used to localize responses evoked by visual, somatosensory and simultaneous bimodal stimuli. Color maps show activation from a typical session, 1 s after the stimulus. Anatomical areas are delineated in white according to Paxinos and Watson [73]. B: Schematic of sensory areas (as in A) together with locations of individual recording sites in parietal (white stars) and visual (yellow circles) cortices. Small insert: localization of the area on the rat brain. C: Left panel: Region of overlapping activation by unisensory stimuli. White region indicates overlap areas with median correlation to both unisensory stimuli. Right panel: Dual-color overlay of visual and somatosensory responses. Intermediate (violet) colors indicate activation by both stimuli. The white cross indicates the multisensory recording location chosen for subsequent electrophysiology. D: Comparison of measured mean hemodynamic signal (blue, mean response) and artificial gamma-function based hemodynamic response (orange, gamma) used for correlation analysis in panel C. The artificial hemodynamic response was constructed from the stimulus pulse train convolved with a gamma probability density function and closely resembles the measured signal time course. E: Fluorescence micrograph of recording location marked by fluorescent dye DiI. The cortical mantle is delineated by the dashed white markings. The right panel shows how the red fluorescence dye has stained the tissue around the electrode trace in the center of the image. Damage from the electrode was minimal. F: Example histological slice from the multisensory parietal region together with a schematic overlay of the multichannel electrode used for recording. Note the morphological characteristics of the association-type cortex with compact layer II and a virtual absence of layer IV. G: Current source density (CSD) analysis of example data (event-related potentials), with current sinks indicated by bright colors (dark areas are equalizing current sources). The strongest activation is located at the depth of the first granular sink (GS, see Results), and an additional sink, likely reflecting direct thalamic input, in layer IV can be seen (*). An infra-granular current sink (IS) and a later supra-granular sink are also visible (SS, falls outside the time window shown for the visual response). The average MUA response (black trace) is overlaid on the CSD data, and shows that both visual and somatosensory stimuli are effective in driving local multi-unit firing. H: Stimulus-response curve from one electrophysiology experiment. The ordinate shows the normalized response amplitude (AVREC) for the probed stimulation intensities denoted on the abscissa. The green rectangle covers the range from which stimuli could be chosen (50 to 90%). The arrow indicates the intensity used in this animal for the main experiments. V1: primary visual cortex, V2: secondary visual cortex, SC: somatosensory cortex, bf: barrel sub-field, PtA: parietal association area, AC: auditory cortex.

For optical imaging, the skull was exposed and thinned over visual and somatosensory areas of the left hemisphere. Thinned skull is sufficiently transparent to record intrinsic optical signals, while also maintaining mechanical stability of the cortical surface, reducing movement artifacts and minimizing cortical damage and edema. A drop of silicone oil (60.000 cSt, Dow Corning Corporation) was applied to reduce glare. The animal was positioned on a vibration isolation table (Minus K Technology) under a macroscope [57], which projected a field of view approximately 7 mm in diameter onto a high-speed CCD camera (Jai TM-6740 GE, Stemmer-Imaging). Homogeneous dark field epi-illumination was provided by a custom-made ring illumination with high-intensity green LEDs (530 nm). Green light was chosen to measure the blood volume signal as suggested by a recent report [58]. The larger magnitude of this signal compared to red light signal further reduces artifacts (note the virtual absence of blood vessel artifacts in Fig. 1A) and minimizes time spent during optical recording. Also note the rapid onset of the signal (Fig. 1D), comparable to the timescale of red light signals. The entire setup was located in a sound-attenuated chamber. Data from the CCD camera was captured with an ActiveX plug-in (ActiveGigE, A&B Software LLC) from Matlab (The MathWorks Inc.) at a resolution of 640×480 pixels and 180-Hz frame rate.

### Electrophysiology and Histology

Following optical imaging to identify multisensory target areas, the silicone oil was removed, and a small incision was made with a sapphire knife in the remaining bone directly above the target area. A silicon microelectrode (one shank with 32 recording sites spaced 50 µm apart, each 400 µm^2^ in area, Neuronexustech Inc.) was inserted via this incision. The back surface of the electrode was coated with the fluorescent carbocyanine dye DiIC_18_ to later localize the recording site in histological slices (Fig. 1E). The laminar positioning of the electrode was controlled by inserting it 1.6 mm below the brain surface. Electric signals were split into local field potentials (LFP, 1–150 Hz) and multi-unit activity (MUA, 0.9–8.8 kHz) before recording (MAP System, Plexon Inc.). LFPs were digitized continuously, and triggered spike data were saved as waveforms and timestamps for offline analysis.

At the end of the experiment, the animal was killed by anesthetic overdose, and the brain was removed and sectioned. Every second section was stained with Nissl stain (Fig. 1F), while the remaining sections were analyzed under a fluorescence microscope to confirm electrode positioning.

### Silencing of Local Activity with Muscimol

To differentiate effects arising from afferent and local activity, local post-synaptic activity was selectively silenced by application of muscimol (4 mM in physiological saline). Muscimol is a potent GABA agonist, which prevents local neuronal spiking by inhibiting all post-synaptic cells expressing GABA receptors. This treatment effectively reduces the contributions of recurrent local connections, and isolates the postsynaptic currents from afferent thalamo-cortical and long range cortico-cortical projections. To prevent nonspecific effects of muscimol on presynaptic GABA(B) receptors, it was paired with the selective GABA(B)-antagonist (+)−5,5-dimethyl-2-morpholineacetic acid (SCH 50911, 6 mM, Schering Pharma) [59]. A small amount (30 µl) of the solution was applied topically to the parietal area within the craniotomy window. The spread of muscimol to the adjacent primary areas was limited by a wall of silicone grease. Based on previous studies, epidural application of muscimol acts locally, with the affected area confined to within about 1 mm of the applied area [60]. While it cannot be excluded that parts of the lower sensory areas are also affected, an influence of the silencing on thalamic nuclei can be safely excluded. Following application, the pharmacological agents were allowed to diffuse into the brain for 1 h, after which almost all spontaneous electrical activity typically ceased. Evoked afferent activity, on the other hand, persisted. For further details of the method, see Happel et al. [61].

### Sensory Stimulation

The visual stimulus was delivered by a 5 mm diameter white LED (Nichia Corp.) placed 1 cm front of the eye contralateral to the studied hemisphere. This created a white disk of approximately 28° visual angle and a luminance of 0.25–2 µW/mm^2^, centered in front of the eye. The surrounding experimentation chamber was dark. A custom-made circuit was used to linearize the brightness response of the LED. Visual stimuli consisted of 1 ms single pulses, with brightness adjusted to avoid saturation of the cortical response (0.25–2 µW/mm^2^). Somatosensory stimulation was provided with a piezo actuator (Physik Instrumente GmbH, Karlsruhe) attached to a plastic mesh, which moved all whiskers of the contralateral whisker pad in the anterior-posterior direction. The mesh did not contact the inter-vibrissal fur, but it is possible that some mechanical coupling between vibrissae and fur was present. Whisker deflections lasted for 1 ms with a typical amplitude of 10–50 µm (approximately 500–2000°/s angular velocity). Target intensities for the somatosensory stimulus were also chosen from the stimulus-response curve to be clearly effective but to avoid response saturation. Adjusted stimuli for each modality elicited 50 to 90% of the maximal response probed across a range of different stimulus intensities and recording modalities (optical imaging: peak amplitude, electrophysiology: mean AVREC in 100 ms window post stimulus). An example is shown in Figure 1H. For the imaging experiments, 3 s duration 10 Hz trains of these stimuli (inter-trial interval: 20 s) were presented. For electrophysiological recording, single pulses were presented. These pulses could be synchronous or asynchronous with delays of 200, 100, and 50 ms in both directions, i.e., visual first or somatosensory first (inter-trial interval randomized from 1 to 2 s, for trials with asynchronous stimuli, inter-trial interval no less than 1.5 s). All stimuli were presented in a pseudo-random order. The difference in stimulation conditions for optical and electrophysiological experiments was necessary to provide a reliable optical signal, as well as identifiable components in the current source density response pattern.

### Data Analysis

Data were analyzed in Matlab. The optical imaging data was low-pass filtered (2 Hz) to remove heartbeat artifacts, mean-adjusted, and down-sampled to 10 Hz. The images were spatially down-sampled to 160×120 pixels to enhance the signal-to-noise ratio. No flat-field correction was performed (division by individual mean pixel intensity) to avoid blood vessel artifacts. For visual inspection (Fig. 1A), activation maps at 1 s post-stimulus onset were computed from trial-averaged responses [58], [62]. Depending on the signal-to-noise ratio of the individual session, 30 to 70 stimulus repetitions were averaged, until the hemodynamic response could be clearly separated from background noise. Two types of quantitative analysis were used to calculate the overlap of unisensory activation patterns. i) Co-activated “multisensory” pixels were defined as those that exceeded the median amplitude in each unisensory condition at 1 s post-stimulus (median calculated across pixels for each condition) [58]. ii) A cross-correlation analysis [63] was used to define active voxels. The stimulus time course was convolved with a normalized gamma probability density function, which approximates a hemodynamic response (Matlab, parameters A: 3 B: 0.4) and provided a good fit to the experimentally measured response time course (Fig. 1D). The activation of each pixel was then defined as the correlation of the pixel time course and this model. Active pixels were defined as those for which the correlation exceeded a threshold defined by the median of all positive correlation values.

Current source densities (CSDs) were defined as the negative second derivative of the local field potential over cortical depth [64], [65] after averaging (n = 300–500 trials) and smoothing with a 350 µm Hanning window, as described by Happel et al. [61]. The CSD of an individual channel 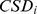 can therefore be approximated by:



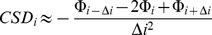
.

Layer localization of the individual sinks was derived from the conserved CSD pattern across sites, and confirmed with histology. The channel providing the current sink of largest amplitude was identified as follows: the strength of the CSD sink (negativity) was determined as average amplitude in a post-stimulus time window chosen to fit the respective duration of unimodal responses (50–100 ms for visual stimuli, 5–25 ms for somatosensory stimuli). The difference in these windows reflects the known response properties of visual and somatosensory responses, with the latter being more transient [66], [67]. To quantify the total activation caused by different stimuli, the average rectified CSD (AVREC) [68], which integrates activity from all layers, was calculated as:
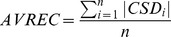



with *n* denoting the number of CSD channels. A window of 100 ms post-stimulus was chosen to represent the total activity elicited by a stimulus. Onset latencies for CSD sinks were calculated as the point where the time course crossed a three-standard-deviation threshold of baseline variability [61].

**Figure 2:**
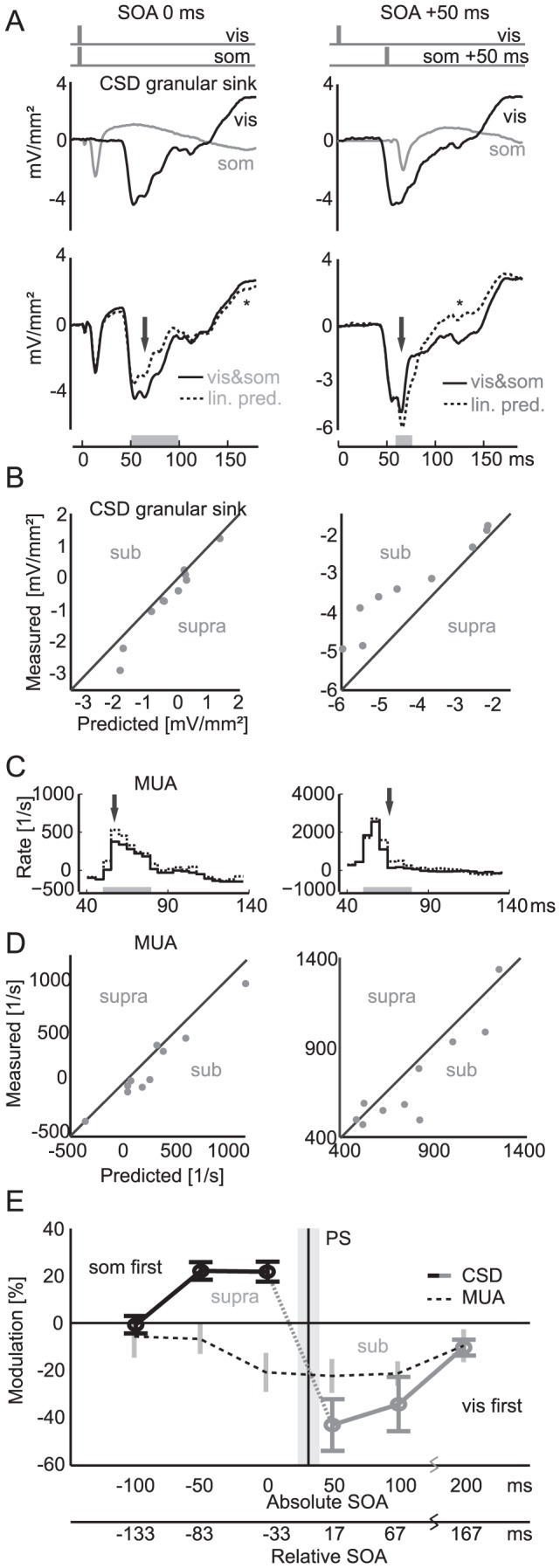
Multisensory response patterns in parietal cortex. A: Example response time course of a granular layer CSD averaged across trials. The upper panel shows responses to each unimodal stimulus, with evoked somatosensory (gray) or visual (black) current sinks (negative values). The lower panel displays the response to the combined stimulus (solid) and the linearly predicted (lin. pred.) response, which is the arithmetic sum of the two unisensory responses (dashed). Here and in panels B, C, and D, the left column shows the responses for a stimulus onset asynchrony of 0 ms (physically synchronous stimuli), while the right panel displays responses for the visual stimulus preceding the somatosensory stimulus by an SOA of 50 ms. In both conditions, the response demonstrates a systematic deviation from the linear prediction, reflecting a non-linear multisensory interaction. For further analysis, we focused on the early part of this interaction (arrow and gray bar) and did not consider effects occurring much longer after stimulus offset (*).B: Distribution of measured and predicted (summed) multisensory CSD responses (strength of granular current sink) in individual experiments (gray dots). Responses to visual stimuli following a somatosensory response (SOA = 0 ms) are supra-linearly enhanced (left), while somatosensory responses following a visual stimulus (SOA = 50 ms) interact sub-linearly (right, the words ‘sub’ and ‘supra’ describe the interaction polarity on their side of the diagonal). More negative values indicate stronger current sinks. C: Example of sub-linear effect on multi-unit activity. The dashed line outlines the sum of unisensory responses, and the solid line represents the measured multisensory response. The gray bar indicates the 30 ms window used to capture the excitatory part of the response. Arrows denote the part of highest non-linearity. D: Distribution of measured and linearly predicted (summed) multisensory MUA responses. Regardless of stimulus onset asynchrony, a sub-linear interaction is observed. E: Multisensory enhancement index (MEI) for the granular CSD sink and MUA over a range of SOAs. CSD responses to visual stimuli preceded by a somatosensory response (black) show a supra-linear effect, while the reverse leads to a sub-linear effect (gray). Note that absolute SOA refers to the external physical SOA of the two stimuli, while relative SOA refers to the internal difference in response latency of the respective unimodal CSD responses. PS indicates the point of simultaneity (mean and s.d.) in individual experiments, at which activity onsets would coincide. Note that the curve is centered around the point of simultaneity and not around synchronous stimuli at an SOA of 0 ms. MUA (dashed line) shows a consistent sub-linear interaction, also restricted to a short time window around simultaneity.

MUA responses were obtained from high-pass filtered signals (0.9–8.8 kHz) as events passing a threshold determined manually for every channel during the experiment. The time course of MUA responses was binned at 5 ms resolution, and the average MUA during a 90 ms pre-stimulus window was subtracted to ensure equal baseline across channels and conditions. The amplitude of MUA responses was quantified in a window of 30 ms and for each experiment, responses were averaged over all channels that demonstrated a stimulus-related increase in firing. The window of 30 ms was chosen because it captured well the excitatory part of the MUA response (the window is depicted as grey bar in Figure 2C). If longer windows are used, the post-response hyperpolarization dependent suppression would be captured as well. Since these components are fundamentally different in nature, we chose to limit the analysis to this first part of the response. We used the same window for both modalities, since the transient excitatory segment of the MUA responses did not show such marked duration differences as seen in the CSD patterns.

Multisensory interactions were quantified by comparing the response to the bimodal stimulus (vis&som) to a prediction obtained by linear summation of the two unisensory responses [69], [70]. Given the linear superposition principle of electrical fields in resistive media, significant deviations from the linear prediction are indicative of supra-linear or sub-linear multisensory interactions. The strength of multisensory interaction was quantified for each signal using the multisensory enhancement index (MEI), which expresses the deviation from the linear prediction relative to the affected unisensory response amplitude [71]: 
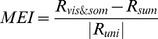



The variables are 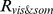, multisensory response; 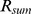, sum of unisensory responses; 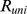, response to the individual unisensory stimulus. The sensory modality of the denominator 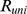
is chosen to be either somatosensory or visual, depending on which response is analyzed. This is necessary because the CSD resolves the individual components of each response, and in particular, because stimuli which are temporally offset require the analysis of the response arriving second. The response to the first stimulus is often not affected by multisensory processes, especially if it occurs even before presentation of the second stimulus. A normalization based on the modality which responded stronger would also be possible, but we decided against this, given that the stronger modality differed from experiment to experiment. Our MEI normalization with the first stimulus' unimodal response is able to compensate for such response magnitude differences between visual and somatosensory responses. This resulting measure is dimensionless. Note that in the presence of purely unisensory neurons, the sum of unisensory responses will match the observed response under multisensory conditions ('no inter-sensory interaction') and therefore the MEI will be zero. Responses are taken as mean values in the time windows, as defined above and indicated by gray bars in the respective figures. In experiments with pharmacological silencing, the peak of the granular sink was used instead of a window average to define response strength, because the signal was too weak to give accurate values over a time window.

## Results

### Localization of a Multisensory Area Using Optical Imaging

We observed clear modality-specific activation in response to unisensory visual or somatosensory stimuli over the respective visual and somatosensory cortices (Fig. 1A, left and middle panels). This activation extended beyond the presumed anatomical borders of the respective cortical areas, in agreement with results of previous studies [34], [35], [72], [73]. Not surprisingly, a continuous area spanning from primary visual to primary somatosensory cortex was activated in response to the combined multisensory stimulus (vis&som; Fig. 1A, right). Given that no region was activated exclusively by the multisensory stimulus, we defined “multisensory” pixels as those that were activated independently by both modalities. Multisensory pixels clustered systematically near the borders of visual and somatosensory cortices in parietal cortex (Fig. 1C). As there is no universally accepted definition of activated area in imaging, we used two different criteria to define co-activated pixels. Both gave similar results. The multisensory region obtained using a median-amplitude threshold extended for 2.1±0.3 mm in the anterior-posterior direction (mean±s.e.m. 1 s post-stimulus, n = 11 experiments). This size was larger than expected on the basis of pure light scattering [58]. The multisensory region obtained using a correlation map had a similar size (2.4±0.3 mm), confirming the existence of an extended multisensory region co-activated over the parietal cortex.

For each experiment, we identified the approximate center of the visual-somatosensory overlap region and selected this area as the target for subsequent electrophysiological recordings. These multisensory “hotspots” are indicated in Figure 1B for individual experiments. The locations were scattered along a narrow strip in the medio-lateral axis, between the visual and somatosensory cortices. This region of strongest multisensory overlap coincides well with the stereotaxic location of parietal association cortex (area PtA) as defined by Paxinos and Watson [73], which is indicated in Figure 1B. We further confirmed this stereotaxic localization with histological analysis. Nissl-stained sections containing the electrode location revealed a compact layer II and very sparse layer IV, a cytoarchitectural structure that is typical for association areas (Fig. 1F) [72]. Overall, these results demonstrate multisensory activation patterns in the parietal cortex of the rat which is conducive to subsequent studies on the neural mechanisms of multisensory processing using electrophysiology.

### Neural Activity in the Parietal Multisensory Region

Electrophysiological recordings confirmed the multisensory nature of this parietal region. Laminar multichannel silicon electrodes were inserted into the cortex at the site of greatest co-activation by visual and somatosensory stimuli, as derived from optical imaging. From these electrodes, we obtained CSDs and multi-unit responses.

CSD analysis revealed both somatosensory and visual inputs in granular and infra-granular layers, with the strongest current sinks (local depolarizing currents) in layers III and IV, most likely reflecting thalamic and intra-cortical inputs (Fig. 1G, bright color) [9], [61], [72]. These sinks were accompanied by compensating sources (dark color) in other layers, which reflect equalizing currents in response to local depolarization at the sink [65]. Dissociating the contributions of layers III and IV to the most prominent early sink is difficult in the rodent, due to the thinner cortical mantle. This difficulty is especially acute in association areas with sparse layer IV, so we refer to this whole joint entity as the "first granular sink" (labeled GS; Fig. 1G) [61]. Based on results of previous studies [61], [74] and the presence of an additional, much weaker sink slightly deeper (0.7 mm below the cortical surface, Fig. 1G), it seems likely that this granular sink reflects feed-forward thalamic input to a lesser extent, but rather reflects a mixture of spread of activity from input to more superficial layers, local cortical amplification, and horizontal cortical afferents [61], [75]. We did not term this sink "supra-granular", despite the prominent involvement of layer III, in order to allow a distinction from more superficial activation that occurred later in the response. Therefore, when comparing this activity to data from a primary cortical area in primates [9], it should also be compared to activity in the lower portions of the supra-granular layers, since an exact match of the CSD patterns between species is often not possible.

In addition to this first granular sink, a weaker infra-granular sink was visible in infra-granular layer V (marked IS in Fig. 1G). This sink was described in previous studies and has also been partially attributed to thalamic input and cortico-thalamic feedback [61]. In the following analyses, we focused on the multisensory interactions in the amplitudes of these granular and infra-granular sinks to reduce data dimensionality.

The multisensory convergence of visual and somatosensory activation within this parietal region was characterized by the typical feed-forward CSD response pattern elicited by both sensory modalities. Visual and somatosensory parietal activation differed in latency, an observation which agrees with known differences in the response latencies between primary visual and somatosensory cortices [66], [76]. Latencies determined from the first granular current sink were shorter for somatosensory (12.6±1.6 ms, mean±s.d.) than for visual stimuli (45.3±8.9 ms, Fig. 2A, t test, *P*<0.01).

The multisensory nature of this parietal region was also exemplified by visual and somatosensory driven multi-unit (MUA) responses. Both modalities evoked spiking responses. MUA responses are indicated in Figure 1G as the average population firing rate across all cortical layers, as only small differences in MUA response shape were observed across layers. The amplitude of MUA responses was somewhat lower in superficial layers, probably reflecting the described sparse firing characteristics of these layers [77].

### Non-linear Response Interactions

While the activation of this parietal region by visual and somatosensory stimuli suggests a multisensory nature, such multisensory convergence or overlap does not necessarily establish the presence of integration of the multisensory responses [78]. To establish true integration, the neural responses to simultaneous multisensory stimuli would have to deviate from the linear summation of the two independent unisensory responses or exceed the strongest unisensory response [79], [80]. To directly probe for such multisensory interactions, we compared CSD and MUA responses of combined vis&som stimuli to the sum of the two unisensory responses (vis+som, R_sum_).

**Figure 3:**
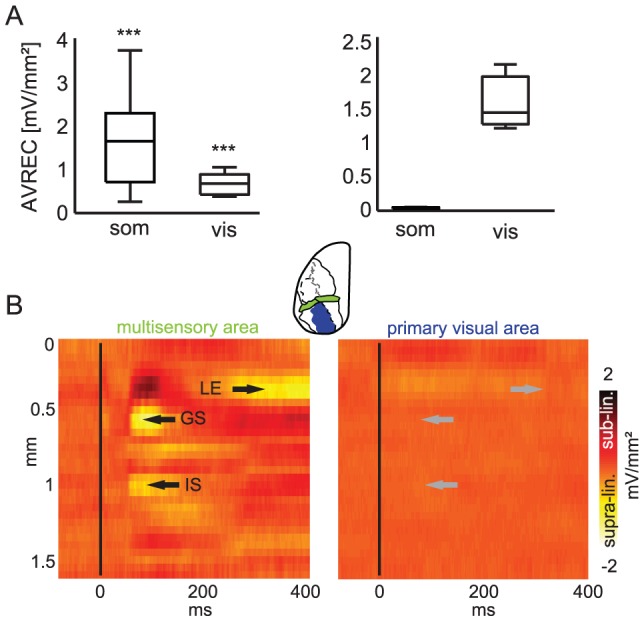
Visual cortex shows less multisensory response. A: The total CSD response to both unimodal stimuli, expressed as averaged rectified CSD (AVREC), confirms the multisensory nature of the investigated parietal region and the near absence of somatosensory-evoked responses in visual cortex. B: CSD responses quantified as the deviation of the measured to the predicted linear multisensory response (SOA = 0 ms, vis&som – (vis+som)). Supra-linear interactions are marked by arrows in the granular (GS) and infra-granular sinks (IS) and also as a late effect in supra-granular layers (LE). Note the absence of multisensory response interactions in visual cortex (right panel, gray arrows mark the same locations as in left panel).

When both stimuli were presented simultaneously (physical stimulus onset asynchrony = 0 ms) the CSD response to the combined stimulus consisted of a leading somatosensory-evoked sink followed by a later visual-evoked sink (Fig. 2A, left), as was expected given the differences in response latencies between the two modalities. Importantly, the somatosensory stimulus (whose activation emerges first) induces a supra-linear enhancement of the amplitude of the visual-evoked current sink; that is, the sink becomes stronger (more negative). This multisensory interaction can be seen in the comparison of the multisensory response and the prediction from the sum of unisensory responses (Fig. 2A, lower panels). The scaled magnitude of this difference is represented by the MEI. The difference between the multisensory response and the unisensory sum was significant across experiments (n = 10, multisensory enhancement index (MEI) = 21.6±4%, mean±s.e.m, Wilcoxon signed rank test: *P*<0.01, Fig. 2B). A similar effect was seen in the infra-granular sink, albeit to a lesser degree: the visual response was enhanced by MEI = 13±13.5% (mean±s.e.m., *P*<0.1), and this enhancement was clearly visible in individual experiments (Fig. 3A). As expected, the amplitude of the somatosensory current sink, which preceded the visual response in time, was not affected (MEI = 6.2±8.2%, *P*>0.9). These results demonstrate a non-linear interaction of visual- and somatosensory-driven responses when the stimuli are presented simultaneously, whereby the somatosensory stimulus enhances the current sink induced by the visual stimulus.

We also found that visual stimuli can affect a subsequent somatosensory response if the unimodal response latencies of both stimuli are accounted for. For example, we performed additional experiments where the somatosensory stimulus was presented 50 ms after the visual stimulus (Fig. 2A, right). Introducing this onset asynchrony effectively caused the visual response to precede the somatosensory response by 17.4±8 ms (mean±s.d.). Similar to the simultaneous condition, we found that the response emerging first in time (here visual) modulated the current sink of the response emerging second (somatosensory) in a manner that deviated from a linear superposition of both responses (Fig. 2A, right lower panel). Unlike in the previous simultaneous condition, however, the amplitude of the somatosensory-evoked granular sink was reduced, i.e., became less negative (MEI = −43±11%; n = 10, *P*<0.005, Fig. 2B). A similar sub-linear interaction was found for the infra-granular sink (MEI = −25.6±13.2%, mean±s.e.m., *P*<0.05). As a control, the amplitude of the visual response (arriving first) was not measurably affected (MEI = −0.4±0.4%, *P*>0.3), although we would like to note that the time interval between the arrival of visual and somatosensory activity, on which this result is based, is short (the first intermediate peak in Fig. 2A, right lower panel, 17.4±8 ms).

In addition to these non-linear interactions during the initial current sinks, we also observed deviations from the linear superposition of unimodal responses at longer post-stimulus latencies. These late effects (Fig. 2A, marked with *) appeared as modulations of supra-granular activity in the superficial 300 µm of cortex. These late responses appeared during periods where they could not be consistently attributed to a well-defined local current sink, and we therefore refrain from further analysis and interpretation. However, these late responses typically shared the same polarity of interaction observed in the preceding granular and infra-granular sinks.

Analysis of MUA response also revealed non-linear multisensory interactions at the level of spiking activity. Overall MUA responses to multisensory stimuli were consistently lower than the sum of the respective unisensory responses, regardless of the sequence of stimulus presentation (i.e. a sub-linear interaction). When the somatosensory preceded the visual response (SOA = 0 ms) visual-evoked MUA amplitudes deviated significantly from the linear prediction (MEI = −20.9±8.3%; *P*<0.05, Fig. 2C,D) and a similar result was obtained when the visual response preceded the somatosensory response (SOA = 50 ms; MEI = −22.4±7.2%; *P*<0.01). These multisensory interactions for MUA did not differ significantly between layers, and all layers shared the same sign of interaction polarity. These results not only confirm the supra-threshold multisensory nature of this parietal region, but also highlight a qualitative difference between spiking and sub-threshold (CSD) activity response patterns. While the sign of multisensory interaction was dependent on stimulus sequence for evoked current sinks, the polarity of the interaction was the same for MUA regardless of stimulus sequence.

### Temporal Properties of Response Interactions

To better characterize the dependency of the multisensory response interactions on stimulus order and timing, we probed these interactions systematically across a wider range of SOAs (Figure 2E). SOA is expressed both in terms of “absolute” SOA with regard to physical stimulus presentation and in terms of “relative” SOA with regard to the latency difference at which the unisensory stimuli induce granular current sinks. In general, this analysis revealed that the magnitude of response interaction decreased with increasing temporal delay between the stimuli, with vanishing interactions for stimuli separated by more than 100 to 200 ms. This neural window of response interaction is similar to behaviorally reported windows of multisensory integration and similar studies of neural activity in other systems [78], [81].

The strongest enhancement of visually evoked CSD sinks was observed when the somatosensory response preceded the visual response by 32.6 ms. On the other hand, the strongest reduction of the somatosensory-evoked sink occurred when the visual response preceded the somatosensory response by 17.4 ms. Hence, our results suggest that the asymmetry in multisensory response interaction is not centered around the point of stimulus synchrony (absolute SOA = 0 ms), but rather around a point close to “cortical simultaneity,” which for the present data is at 32.6±8 ms absolute SOA (interpolated, mean±s.d., black line in Fig. 2E). Notably, this time point corresponds to the difference in response latencies between the two unisensory conditions (approximately 33 ms, see above; rel. SOA 0 ms), suggesting that the sign of multisensory interaction in CSD sinks depends on the relative order with which both unisensory responses activate local parietal networks.

In contrast to CSDs, multisensory interactions in MUA responses were independent of stimulus order. This observation suggests that multisensory interactions in stimulus-evoked current sources do not translate one-to-one into similar interactions at the level of neural spiking activity. Rather, different signs of multisensory interactions appear in supra- and sub-threshold activity depending on the stimulus sequence presented. The sub-linear multisensory interactions in spiking activity observed here may be partly a consequence of using an aggregate measure of activity (MUA) rather than single neuron responses; however, the sub-linearity agrees well with results from other methods, including single neuron recording [10]–[12], [82], [83].

### Comparison with a Primary Area

To confirm that these multisensory response patterns are specific to the parietal multisensory region, we recorded additionally from primary visual cortex (V1; n = 3), and compared both unisensory and multisensory responses to the parietal area. This test was performed using the SOA = 0 ms condition, for which the somatosensory response emerges before the visual response in parietal cortex (see above) and before the visual response in V1 (latency approx. 20 ms).

Figure 3 displays example data from two experiments directly contrasting response patterns in the parietal area and V1. In contrast to the parietal area, which exhibited significant convergent responses to stimuli of both modalities, V1 showed predominantly visual responses. Figure 3A displays the rectified total CSD response (AVREC) to either unisensory condition in both areas. Parietal cortex was co-activated by both stimuli (AVREC somatosensory: 1.8±1.2 mV/mm^2^, t test vs. zero, *P*<0.001; visual: 0.8±0.5 mV/mm^2^, *P*<0.001, mean±s.d., 0 to 100 ms post-stimulus). Visual cortex, by contrast, was activated only by visual stimuli (n = 3; visual: 1.6±0.5 mV/mm^2^), while the somatosensory-evoked response was very weak (0.04±0.02 mV/mm^2^, Fig. 3 A, right panel). In addition to this lack of multisensory convergence, we did not observe any clear deviations from a linear response model. The graphs in Figure 3B display the deviation of the multisensory response from the linear prediction based on the two unisensory responses (i.e., the difference vis&som – (vis+som)). In the parietal area, a characteristic pattern of multisensory responses emerged, including the enhancement of granular (denoted GS in Fig. 3B, left panel) and infra-granular sinks (IS) as well as the later effect in upper cortical layers (LE). By contrast, no deviation from the linear model was evident in visual cortex (Fig. 3B, right; note that both graphs are on the same scale). Rather, the response to multisensory stimulus closely matched the superposition of unisensory responses, demonstrating that potential multisensory response interactions are small (MEI for granular sink: −4.7±2.2%, n = 3). These results show that the reported multisensory response pattern is specific to the parietal area, and does not occur in a primary sensory area such as V1.

### Pharmacological Silencing

Cortical CSD activity is a mixture of afferent activities from subcortical structures, horizontal cortical input, and local processing. The observed multisensory response interactions can emerge from any of these inputs, which makes their interpretation difficult [59], [81]. While CSDs in general allow a separation of current sinks from supra-granular and infra-granular regions, the specific CSD patterns observed here made it difficult to strictly separate between thalamic afferents to the granular layers and cortical afferents to supra-granular layers (this is possibly a result of the specific cytoarchitectural structure of this region, or of the rodent cortex in general). To better elucidate the origin of multisensory response interactions, we performed additional pharmacological tests.

**Figure 4:**
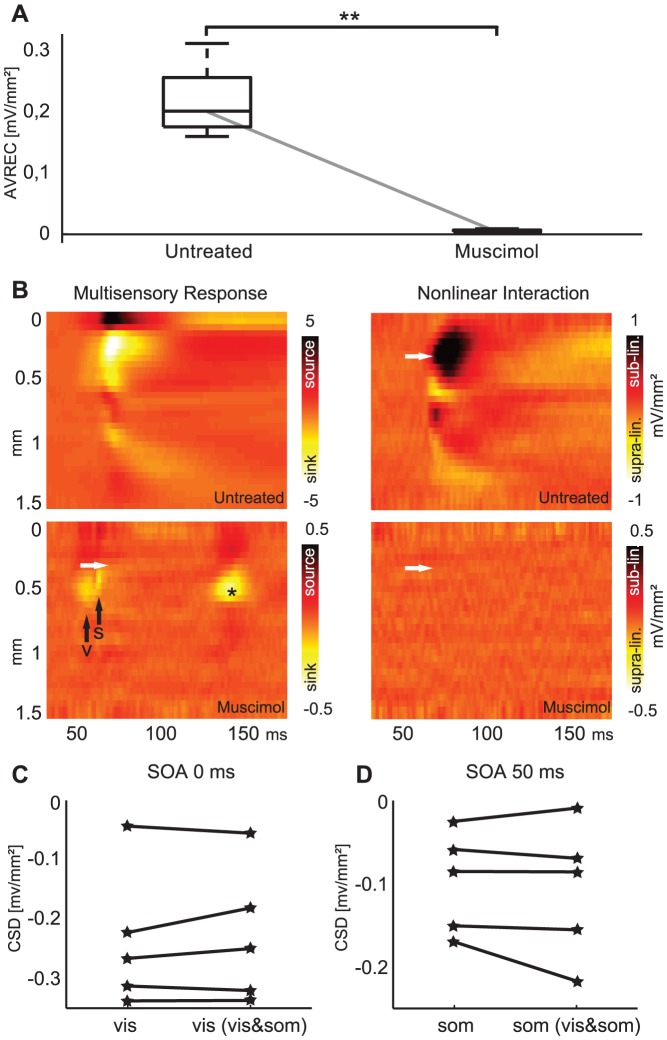
Pharmacological suppression of local activity abolishes non-linear interactions. A: General decrease in average rectified current source density (AVREC) caused by muscimol application (100 ms post-stimulus window, mean of both unisensory stimuli, n = 5, *P*<0.05, U test). B: Example data showing the bimodal response (vis&som) and the non-linear interaction (vis&som – (vis+som)) for the SOA = 50 ms condition before (upper panel) and after application of muscimol. In the untreated condition, the previously noted pattern of current sinks (left) and sub-linear multisensory interactions are visible (white arrow, right panel). After muscimol application, individual feed-forward current sinks related to visual (arrow V) and somatosensory (arrow S) inputs are apparent, as well as a late additional visual sink (*, bottom left panel). The multisensory interaction is negligible, indicating a linear superposition of responses. Note the different scales in upper and lower panels. C: Peak amplitudes of the visual granular sink during unisensory (vis) and multisensory stimulation (vis (vis&som)) after muscimol treatment do not differ, demonstrating the absence of non-linear interactions. D: Peak amplitudes of the somatosensory granular sink in unisensory (som) and multisensory stimulation (som (vis&som)) after treatment also do not differ, favoring a cortical origin of the observed interactions.

We applied the GABA-agonist muscimol in combination with the specific GABA_B_-antagonist SCH50911 to suppress local action potential generation by inducing post-synaptic inhibitory currents (n = 5 experiments). This treatment blocks spiking in all exposed neural elements, except in those originating from outside the treated area. It thereby cancels the contribution of local recurrent connections to the observed CSD patterns. Hence, only afferent synaptic inputs contribute to the CSD. Indeed, the overall CSD response strength as quantified by the AVREC in a 100-ms post-stimulus window (averaged across visual and somatosensory stimuli) was greatly reduced following muscimol application (before treatment: 0.22±0.03 mV/mm^2^ mean±S.E.; after treatment: 0.0053±0.0012 mV/mm^2^, U test *P*<0.01, Fig. 4A). This finding suggests that only 2–3% of the measured activity originates from currents caused by afferent synapses. While this number appears small, one must keep in mind that thalamocortical projections to, for example, the visual cortex account for a similarly small percentage of synapses [84], [85]. Furthermore, the number cortical inter-areal projection synapses is considered to be very small compared to local synapses [86]. Since it is difficult to control the spread of muscimol in the tissue of such a small area, it appears likely that these remaining synapses are mainly thalamocortical and that certainly a number of the intra-cortical connections– if not most – have also been silenced. Nonetheless, this experiment demonstrates that the multisensory effect is not already contained in thalamic input.

Figure 4B displays a typical CSD response for the SOA = 50 ms condition on the left column, before and after muscimol silencing. After muscimol application, CSD amplitude decreases overall. In addition, multisensory response interactions disappear, with clear visual and somatosensory related components remaining (black arrows on lower left panel of Fig. 4B, and lack of interaction in lower right panel). The two remaining components combine linearly, i.e. the total activity is completely explained by summing individual unisensory activations. This linearity of the two remaining components after silencing is further illustrated in Fig. 4C and D. The part of the multisensory CSD response pattern of either modality has approximately the same value as if the respective unisensory stimulus were presented. Disappearance of sub-linear interaction was observed in all muscimol application experiments (n = 5) and for different sequences of unisensory stimuli (i.e., SOA = 0 ms and SOA = 50 ms conditions). The associated MEIs were also small and not significantly different from zero following treatment (1±7.5%, SOA = 0ms; −2.8±15.9%, SOA = 50 ms; Wilcoxon rank-sum test, *P*>0.05). This shows that the described non-linearities are not relayed by thalamic afferents but likely arise in the local population activity generated by the arrival of the first sink.

The pharmacological treatment also provided additional insights into the laminar structure and timing underlying the multisensory interaction. The onset of the non-linear response interaction in the untreated cortex appeared when the afferent unisensory activity (as determined from the muscimol treated preparation) had ceased. This phenomenon can be seen in Figure 4B by comparing the white arrow (onset of interaction) with the black arrows (onsets of unisensory responses) and suggests that sustained activity emerging from local networks is critical for the non-linear and stimulus sequence-dependent response interactions. Note that this sustained activity was absent in the muscimol-treated cortex, which exhibited only brief stimulus-evoked responses. In addition, muscimol application also unmasked a slightly more superficial localization of the somatosensory granular sink compared to the visual granular sink (Fig. 4B, lower right). Across animals, the peaks of these sinks differed by 150±32 µm (*P*<0.01, U test). This difference in laminar response patterns may contribute to the asymmetry of CSD interactions. Additionally, muscimol application also revealed a late visual input, approximately 140 ms post-stimulus (Fig. 4B, marked with star). While irrelevant for the early interactions analyzed here, this observation highlights a potential second source of visual inputs to parietal cortex that may be worth future investigation. Taken together, these results strongly suggest intra-cortical networks as the predominant source for the observed non-linear interactions and provide conclusive evidence against immediate subcortical origins.

## Discussion

Our results highlight multisensory response properties of the rat parietal cortex. We found a convergence of visual and somatosensory responses in a region corresponding to the anatomically defined area PtA, and we found non-linear interactions between visual- and somatosensory-evoked responses in sub-threshold (current sinks) and supra-threshold (MUA) activity that were dependent on the relative timing of the individual stimuli. These findings characterize this parietal region as bimodal (sensory convergence) and as featuring key functional criteria typically associated with multisensory integration [1], [10], [33], [78]. Control experiments demonstrated the absence of such multisensory interactions in primary visual cortex, and thereby rule out trivial explanations such as response modulations due to anesthesia effects or global neuromodulatory influences. We provide in this report a detailed localization of this visual-somatosensory region and demonstrate the cortex-dependent, asymmetric, and timing-dependent nature of this interaction. In contrast to previous work by Barth and colleagues [50], [67] who described a parieto-temporal region responsive to auditory and somatosensory stimuli, the region reported here is located further posteriorly and more medially. It is also distinct from the audio-visual region described by Hirokawa and colleagues which is located in the lateral parts of secondary visual cortex [87].

Experiments involving stimulation of more than one sensory system are more vulnerable to the effects of variable brain states. This problem was minimized through the use of urethane anesthesia, which is known to preserve localized sensory-evoked responses and which, in contrast to other anesthetics, does not induce sensory-driven bursts of activity that propagate far from the source [88], [89]. Nonetheless, it will be interesting to examine what influence urethane has on the laminar response patterns. It is possible, for example, that the supra-granular layers are even more involved in the awake state, which would explain differences to other studies [9]. Awake preparations may also be more amenable to the analysis of the oscillatory contributions to multisensory integration [9].

### Visual-somatosensory Interactions in Parietal Cortex

We found that multisensory response interactions emerge rapidly following stimulus onset and modulate the strength of stimulus-evoked current sources in supra- and infra-granular layers. The laminar pattern of multisensory interactions indicates an effect resulting mainly from intracortical connections. Indeed, anatomical studies demonstrate the presence of lateral cortical input from primary sensory cortex to parietal regions in these layers [72], and functional studies examine cortical contributions to granular CSD patterns [61]. Upon pharmacological silencing of local post-synaptic activity, non-linear multisensory interactions vanished. This finding supports the view that the observed interactions of visual- and somatosensory-driven activation result from intra-cortical processes and do not simply mirror or relay multisensory interactions at thalamic stages. While the diffusion characteristics of muscimol make it prone to also affect parts of the adjacent sensory cortical areas, its diffusion characteristics are sufficiently local to safely exclude subcortical effects. In future studies, optogenetic inhibition of this area may be able to provide a more localized action. Nonetheless, by combining the temporal characteristics of the effects, their laminar profile and the results of pharmacological silencing, our results strongly suggest the critical involvement of local intra-cortical processes in the observed response interactions.

Our main goal was to localize multisensory responses within parietal cortex (using imaging) and to directly demonstrate the convergence and interaction of multisensory inputs at the network level (CSD analysis). In addition, our analysis of multi-unit spiking responses demonstrates the resulting impact of multisensory stimuli at the neural ensemble level. Whereas CSD sinks exhibited supra-linear or sub-linear response interactions depending on stimulus timing (i.e. corresponding deviations for a linear response superposition), multi-unit responses were consistently reduced during multisensory stimulation compared to the sum of unisensory responses. This observation deviates from the classical concept of supra-linear multisensory response enhancement, based on early studies in the superior colliculus [78]. This may have several reasons. Most importantly, MUA does not reflect a single neuron's response, and hence is confounded by the inclusion of different unisensory and multisensory responses into a single index [38]. It is therefore conceivable that this might lead to an inherent bias towards sub-linear response interactions when using MUA to index multisensory processing, similar to related concerns when using the aggregate fMRI-BOLD signal to study multisensory processing [79] (see also [80]). An additional caveat of interpreting MUA responses concerns simultaneous spiking, in which case MUA can show artifactual sub-linearity, as some simultaneous spikes are not accurately detected. In our experiments – due to the different response latencies – peak responses are typically non-overlapping, and we therefore do not consider this to be a major factor. To conclude, sub-additive multisensory response interactions in MUA do not conclusively extrapolate to individual neurons, nor does it rule out the existence of true multisensory-enhanced neurons. A sensible prediction is that the described multisensory region contains both unisensory neurons responding only to visual and somatosensory stimuli as well as a subset of truly bimodal neurons, that possibly show patterns of multisensory enhancement or suppression, as reported recently in a ferret parietal area [38], [37].

The finding of such sub-linear population level effects might also point to a prominent role of inhibition in cortical multisensory processing, a hypothesis that is fostered by recent studies in the cat [90], intracellular recordings in mice [91], and computational models replicating cortical multisensory response patterns [82]. Given the prominence of recurrent trans-laminar inhibition in cortical microcircuits [75] and given that our results suggest an intra-cortical source for the response interaction, it would not be surprising if inhibitory interneurons played a central role in shaping the multisensory interactions.

### Stimulus Order and Timing-dependent Interactions

An intriguing result of our experiments is the temporal asymmetry of visual-somatosensory interaction measured in granular current sinks. While a preceding somatosensory response enhanced the current sink evoked by a visual stimulus, suppression occurred when the visual response preceded the somatosensory current sink. This surprising finding suggests an asymmetry in the underlying pathways or networks engaged by the respective signals. Indeed, our pharmacological tests revealed slight differences in the cortical depths of visual-evoked and somatosensory-evoked current sinks, whereby visual responses were located deeper than somatosensory responses. Additional studies are required to elucidate the specific circuits activated by each modality in detail; for example, using a combination of genetic labeling and functional imaging techniques [23], [92]. A speculative explanation could be that visual and somatosensory inputs target distinct microcircuits whose activation induces subsequent network activity with different levels of excitation-inhibition balance.

An interesting consequence of such processes is the minimal modulation at the point of simultaneity itself, where facilitation changes to suppression. In this case, simultaneous activity leads to a new balance of the enhancing and suppressive processes such that their resulting extracellular currents cancel each other out and lead to a condition in which no net interaction can be detected by CSD or LFP measurements. However, this special condition should not imply that response interactions cease–rather, their opposing polarity may hide interactions from observation. We would like to note in this regard, that the CSD analysis is particularly susceptible to currents arising from "open fields", that is spatially separated influx and efflux of current [65]. Typically, only a subset of cortical cells–mainly the pyramidal cells of layer II/III with large and parallel dendrites–generates such fields. Our low-impedance MUA recordings, on the other hand, target a much larger set of different cells, many in the deeper layers, which could account for the observed differences in multisensory interaction polarity between MUA and CSD analysis. One remaining question concerns the temporal properties of response interaction at a finer time scale. As shown by the Stein lab [53], at least in the superior colliculus, there exists a special relationship between the inhibitory part of a neuronal response and the magnitude of response modulation. It remains to be seen in future work, whether the same is true for this particular cortical system as well, or whether strong modulations exist already during the excitatory part. Such a study would have to employ sensory stimulation at millisecond temporal resolution very close to the point of cortical simultaneity.

The discovered functional asymmetry bears intriguing similarities to perceptual asymmetries reported for the detection of stimuli presented to other modalities. For example, the well-known Colavita effect [93], [94] describes an asymmetry between sensory modalities in competing for attention or processing resources, such as when human subjects fail to detect auditory targets accompanied by visual stimuli. This effect has been taken to suggest a dominance of one modality over another, at least under certain experimental conditions. Whether the observed response asymmetry is related to similar behavioral asymmetries in the rat remains an interesting question, especially since the rat is often regarded as a less visual animal. The fact that the visual activity is enhanced with interaction–whereas somatosensory activity is suppressed–provides support for an alternative view [24].

When considering the functional relevance of visual-somatosensory interactions, it is important to keep in mind that the time scales of typical stimuli in the rat visual and somatosensory modalities differ. While visual information is informative of global information about the environment or social interactions [95], whisker-related information relates to object contact in the immediate surround and changes on a very fast time scale [24], [96]. Therefore, in the rat, visual information likely provides a “context” within which whisker-related information is interpreted. With this in mind, we suppose that the visual-first condition is the more relevant for rodent behavior. Our results show that in this condition, visual stimuli reduce current sinks and spiking activity evoked by a somatosensory stimulus. How this phenomenon relates to potential behavioral patterns of sensory integration remains to be tested.
